# Outer Membrane Vesicles Derived From *Escherichia coli* Regulate Neutrophil Migration by Induction of Endothelial IL-8

**DOI:** 10.3389/fmicb.2018.02268

**Published:** 2018-10-11

**Authors:** Jaewook Lee, Yae Jin Yoon, Ji Hyun Kim, Nhung Thi Hong Dinh, Gyeongyun Go, Sookil Tae, Kyong-Su Park, Hyun Taek Park, Changjin Lee, Tae-Young Roh, Dolores Di Vizio, Yong Song Gho

**Affiliations:** ^1^Department of Life Sciences, Pohang University of Science and Technology, Pohang, South Korea; ^2^Division of Integrative Biosciences and Biotechnology, Pohang University of Science and Technology, Pohang, South Korea; ^3^Division of Cancer Biology and Therapeutics, Samuel Oschin Comprehensive Cancer Institute, Cedars-Sinai Medical Center, Los Angeles, CA, United States

**Keywords:** outer membrane vesicles, extracellular vesicles, exosomes, neutrophil, pulmonary inflammation, IL-8, NF-κB, toll-like receptor 4

## Abstract

Outer membrane vesicles (OMVs) are spherical, proteolipid nanostructures that are constitutively released by Gram-negative bacteria including *Escherichia coli*. Although it has been shown that administration of *E. coli* OMVs stimulates a strong pulmonary inflammatory response with infiltration of neutrophils into the lungs *in vivo*, the mechanism of *E. coli* OMV-mediated neutrophil recruitment is poorly characterized. In this study, we observed significant infiltration of neutrophils into the mouse lung tissues *in vivo*, with increased expression of the neutrophil chemoattractant CXCL1, a murine functional homolog of human IL-8, on intraperitoneal administration of *E. coli* OMVs. In addition, OMVs and CD31-positive endothelial cells colocalized in the mouse lungs. Moreover, *in vitro* results showed that *E. coli* OMVs significantly increased IL-8 release from human microvascular endothelial cells and toll-like receptor (TLR)4 was found to be the main component for recognizing *E. coli* OMVs among human endothelial cell-associated TLRs. Furthermore, the transmigration of neutrophils was suppressed in the lung tissues obtained from TLR4 knockout mice treated with *E. coli* OMVs. Taken together, our data demonstrated that *E. coli* OMVs potently recruit neutrophils into the lung via the release of IL-8/CXCL1 from endothelial cells in TLR4- and NF-κB-dependent manners.

## Introduction

Although *Escherichia coli* and other Gram-negative bacteria are normal flora in the human colon, they can induce sepsis through robustly activating the host immune system (Costerton et al., 1974; Annane et al., 2005; O’Hara and Shanahan, 2006). Sepsis-involved Gram-negative bacteria, such as *E. coli, Pseudomonas aeruginosa*, and *Acinetobacter baumannii*, can secrete outer membrane vesicles (OMVs) (Lee et al., 2007; Kwon et al., 2009; Park et al., 2010; Choi et al., 2011). OMVs are spherical bilayered nanovesicles with diameters ranging from 20 to 200 nm, and are composed of outer membrane proteins, periplasmic proteins, lipopolysaccharides (LPS), nucleic acids, and other virulence factors (Beveridge and Kadurugamuwa, 1996; Horstman and Kuehn, 2002; Kuehn and Kesty, 2005; Lee et al., 2016). OMVs elicit host immune responses by activating several sentinel cells and inducing the release of cytokines/chemokines, further promoting recruitment of inflammatory cells to the inflamed tissues (Zhang et al., 1997; Opal, 2007). Collectively, OMVs released by Gram-negative bacteria are considered to play key roles in sepsis pathogenesis (Park et al., 2010; Kim O.Y. et al., 2013).

Recent reports demonstrated that *E. coli* OMVs induced systemic inflammatory response syndrome (SIRS), characterized by systemic and pulmonary inflammation (Park et al., 2010; Kim J.H. et al., 2013; Jang et al., 2015). On intraperitoneal administration, *E. coli* OMVs are distributed to the whole mice and are accumulated in the lungs within 3 h (Jang et al., 2015). In addition, *E. coli* OMVs induce dysfunction of the lungs by attracting leukocytes, especially neutrophils, and increasing lung permeability and the release of cytokines in the lung tissues (Park et al., 2010; Kim J.H. et al., 2013). During lung injury, circulating neutrophils pass through the endothelial barriers, and transmigrate into the lung tissues (Wagner and Roth, 2000; Craig et al., 2009). Attracted by chemokines, circulating neutrophils first adhere to the endothelium and then transmigrate out of the vasculature into the interstitial tissues (Smith et al., 1991).

In Gram-negative bacterium-associated sepsis, endothelial cells play key roles in sensing the pathogens and recruiting leukocytes to the infected sites (Andonegui et al., 2003, 2009; Harari et al., 2006; Zhou et al., 2009). Although endothelial cells function as the primary barriers to OMVs, the mechanisms underlying OMV-induced modulation of endothelial cells to cause adhesion and transmigration of neutrophils are not fully understood. Recently, our group reported that *E. coli* OMVs induced upregulated expression of cell adhesion molecules in endothelial cells, facilitating neutrophil adhesion to endothelial cells (Kim J.H. et al., 2013). In addition to neutrophil adhesion, endothelial cells can produce neutrophil chemoattractants, such as IL-8 and CXCL1, with consequent transmigration of circulating neutrophils to the inflammatory lesions (Smith et al., 1991; Mohsenin et al., 2007). Endothelial cells, when stimulated with TNF-α, IL-1β, and LPS, secrete IL-8, resulting in transendomigration of neutrophils following the increasing gradient of IL-8 concentration (Huber et al., 1991; Wagner and Roth, 2000). Furthermore, endothelial cells stimulated with cytokines or LPS present IL-8 on the luminal surface to promote neutrophil adhesion (Huber et al., 1991; Middleton et al., 1997). Collectively, OMVs increase endothelial cell adhesion molecules to regulate adhesion of neutrophils (Kim J.H. et al., 2013). However, how these OMVs produce endothelial IL-8 to modulate transmigration of neutrophils is still unknown.

In this report, we provide evidence that *E. coli* OMVs, administered intraperitoneally, can mediate expression of a neutrophil chemoattractant CXCL1 (a murine functional homolog of human IL-8) (Mohsenin et al., 2007; Hol et al., 2010), and neutrophil transmigration into the lung tissues *in vivo*. To elucidate the detailed mechanisms of IL-8 induction, we used several cell types and diverse types of OMVs derived from Gram-negative bacteria. In addition, the roles of toll-like receptors (TLRs) and downstream signaling pathways in IL-8 release from endothelial cells were assessed *in vitro*. Finally, we determined the roles of TLR4 in OMV-induced neutrophil transmigration *in vivo*.

## Materials and Methods

### Mice

This study was carried out in accordance with the recommendations of the Institutional Animal Care and Use Committee at Pohang University of Science and Technology, Pohang, South Korea. The protocol was approved by the Institutional Animal Care and Use Committee at Pohang University of Science and Technology (Approval number: 2011-01-0022). Wild-type and TLR4 knockout mice of the C57BL/6 background were purchased from Jackson Laboratories (Bar Harbor, ME, United States), and 6- to 8-week-old male mice were used for experiments.

### Cell Culture

Human immortalized microvascular endothelial cells (HMEC-1s) and primary microvascular endothelial cells (HMVECs) were cultured in EGM-2 medium (Lonza, Walkersville, MD, United States) (Kim et al., 2002). Human lung adenocarcinoma (A459), bronchial epithelial cells (BEAS2B), monocytes (THP-1 and U937), and T cells (Jurkat and MOLT-4) were cultured in Roswell Park Memorial Institute 1640 (RPMI1640; Invitrogen, Carlsbad, CA, United States) supplemented with 10% FBS, 100 units/mL penicillin, and 0.1 mg/mL streptomycin. Mouse macrophages (RAW264.7) and fibroblasts (NIH-3T3) were cultured in Dulbecco’s modified Eagle medium (DMEM; Invitrogen) supplemented with 10% FBS, 100 units/mL penicillin, and 0.1 mg/mL streptomycin. All cells were cultured at 37°C with 5% CO_2_ in a humidified incubator. All cells were confirmed to be mycoplasma-free, using the e-Myco^TM^ Mycoplasma PCR Detection Kit (iNtRON Biotechnology, Inc., Seongnam, South Korea).

### Isolation and Characterization of OMVs

*Escherichia coli* was obtained from the peritoneal lavage fluids of mice operated with cecal ligation and puncture (Park et al., 2010), and *P. aeruginosa* PAO1 and *A. baumannii* ATCC 15150 were purchased from American Type Culture Collection (ATCC; Manassas, VA, United States). The bacteria were grown in lysogeny broth (Merck, Darmstadt, Germany) at 37°C with gentle shaking (200 rpm) until *A*_600_ = 1.5. The bacterial OMVs were purified from the bacterial culture as described previously, with some modifications (Park et al., 2010, 2013; Kim J.H. et al., 2013). The bacterial cells were centrifuged twice at 5,000 × *g* for 15 min at 4°C. The supernatants were filtrated with a 0.45 μm pore-sized filter, and the filtrates were concentrated using ultrafiltration using a QuixStand Benchtop System (GE Healthcare, Piscataway, NJ, United States) having a 100 kDa hollow-fiber membrane (GE Healthcare). The concentrates were further filtrated with a 0.22 μm pore-sized filter, removing any remaining cells. OMVs were isolated by ultracentrifugation of the filtrate at 150,000 × *g* for 3 h at 4°C and resuspended in phosphate-buffered saline (PBS). To further purify *E. coli* OMVs using buoyant density gradient ultracentrifugation, *E. coli* OMV pellets, resulting from ultracentrifugation of the filtrate at 150,000 × *g* for 3 h at 4°C, were resuspended in 50% iodixanol. The resuspended OMVs were applied to the bottom of density gradients (10 and 40% iodixanol) and subjected to buoyant density gradient ultracentrifugation at 200,000 × *g* for 2 h at 4°C. The fraction containing the purified OMVs were collected from the third fraction from the top layer and subjected to ultracentrifugation at 150,000 × *g* for 3 h at 4°C. The final pellets were resuspended in PBS. The protein concentrations of bacterial OMVs were quantified by the Bradford assay (Bio-Rad Laboratories, Hercules, CA, United State). OMVs diluted in PBS were aliquoted and stored at -80°C until use, as previously reported (Park et al., 2010).

The isolated OMVs were absorbed to glow discharged and carbon-coated 300-mesh copper grids (Electron Microscopy Sciences, Matfield, PA, United States). The OMV-absorbed grids were washed with deionized water and negatively stained with 2% uranyl acetate (Ted Pella, Redding, CA, United States). Electron microscopy was performed with a JEM1011 microscope (JEOL, Tokyo, Japan), at an accelerating voltage of 100 kV.

The size distribution of *E. coli* OMVs was measured by dynamic light scattering with Zetasizer Nano ZS (Malvern Instruments, Malvern, Worcestershire, United Kingdom). Protein samples from whole cell lysates and OMVs (5 μg in total protein amounts) were analyzed using sodium dodecyl sulfate-polyacrylamide gel electrophoresis (SDS–PAGE; 10% resolving gel), followed by gel staining with Coomassie Brilliant Blue R-250 (Sigma-Aldrich, St. Louis, MO, United States).

Protein samples from whole cell lysates and OMVs (1 or 5 μg in total protein amounts for detecting OmpA or FtsZ, respectively) were subjected to SDS–PAGE (10% resolving gel) and transferred to a polyvinylidene difluoride membrane. After blocking with 5% skim milk, the membrane was incubated with a rabbit anti-OmpA (lab-made) or anti-FtsZ IgG polyclonal antibody (R&D Systems, Minneapolis, MN, United States), followed by goat anti-rabbit IgG (Santa Cruz Biotechnology, Santa Cruz, CA, United States), which were conjugated with horseradish peroxidase. A chemiluminescent substrate (iNtRON Biotechnology Inc.) was used to visualize immunoreactive bands.

The vesicular LPS was purified from *E. coli* OMVs (20 μg in total protein amounts) using an LPS extraction kit (iNtRON Biotechnology Inc.), according to the manufacturer’s instructions. The purified vesicular LPS was quantified with the purpald assay (Lee and Tsai, 1999).

### *E. coli* OMV-Induced SIRS Model

Systemic inflammatory response syndrome was induced in mice by *E. coli* OMVs, as previously described with some modifications (Park et al., 2010). Five animals were used in each group. Briefly, *E. coli* OMVs (15 μg in total protein amounts) were intraperitoneally introduced into mice. At 3 or 6 h after the introduction of OMVs, a catheter was inserted into the trachea of anesthetized mice. Bronchoalveolar lavage (BAL) fluids were obtained by introducing and aspirating two successive volumes of PBS 1 mL and pooling these two volumes. After centrifugation at 2,000 × *g* for 10 min, the BAL fluid supernatants were stored at -80°C until use. The lungs were retrieved after whole body perfusion.

### Immunohistochemistry in the Lung Tissues

The harvested lungs were fixed with 4% paraformaldehyde and embedded in paraffin. The embedded lung tissues were sectioned (4 μm thickness), and deparaffinized. The Target Retrieval Solution (DAKO, Glostrup, Denmark) was used for antigen unmasking, and the non-specific binding was blocked with the Protein Block Serum-Free blocking solution (DAKO). The lung tissue sections were incubated with primary antibodies: rat anti-NIMP-R14 (Abcam, Cambridge, United Kingdom), goat anti-CD31 (Santa Cruz Biotechnology), rabbit anti-SP-C (Santa Cruz Biotechnology), or 200 ng/mL of lab-made rabbit IgG polyclonal anti-*E. coli* OMVs (Jang et al., 2015). After treating with secondary antibodies conjugated with fluorescent Alexa Fluor dyes (Molecular Probes, Eugene, OR, United States), the lung tissues were counterstained with Hoechst 33258 (Sigma-Aldrich). Representative images were photographed using an FV1000 Olympus confocal microscope (Olympus, Tokyo, Japan) equipped with FV1000-ASW 3.0 software (Olympus). The number of neutrophils per field was measured by counting random fields in fluorescent images from each lung specimen.

### Real-Time RT-PCR

RNA was isolated from HMEC-1 treated with PBS or *E. coli* OMVs (0.5 ng/mL) for 12 h, using the RNeasy Mini Kit (QIAGEN, Hilden, Germany), following the manufacturer’s protocol. Total RNA (100 ng in total amounts) was subject to amplification with the One Step SYBR RT-PCR Kit (Takara Bio Inc., Kusatsu, Japan) using the LightCycler 2.0 PCR System (Roche Diagnostics, Basel, Switzerland). Primers were designed using the Primer3 program (Untergasser et al., 2012), and the primers used for real-time RT-PCR are shown in **Supplementary Table 1**. Amplification was performed by heating the samples at 50°C for 2 min, then at 95°C for 10 min, followed by repeating cycles of 95°C for 15 s, 55°C for 10 s, and 72°C for 10 s, for a total of 45 cycles. The comparative Ct method was employed to relatively quantify the expression of target genes against that of a house-keeping gene GAPDH (Livak and Schmittgen, 2001).

### Induction and Measurement of IL-8 or CXCL1

To investigate which cell types were the main functional targets of OMVs, various human (A549, BEAS2B, HMEC-1, HMVEC, THP-1, U937, Jurkat, and MOLT-4) and mouse cells (RAW264.7 and NIH-3T3) were plated (1 × 10^5^ cells/well) and treated with *E. coli* OMVs (0.5 ng/mL in total protein concentration) in the presence of 5% FBS at 37°C for 12 h. HMEC-1 were also treated with OMVs (0.5 ng/mL in total protein concentration) from other Gram-negative bacteria (*P. aeruginosa* and *A. baumannii*) in the presence of 5% FBS at 37°C for 12 h.

To study the effects of TLR agonists on endothelial cells, human TLR agonists (InvivoGen, San Diego, CA, United States) or *E. coli* OMVs (0.5 ng/mL in total protein concentration) were treated to HMEC-1 (1 × 10^5^ cells/well) in the presence of 5% FBS at 37°C for 12 h at the following concentration: TLR1/2 agonist, Pam_3_CSK_4_, 100 ng/mL; TLR2/6 agonist, FSL1, 100 ng/mL; TLR2 agonist, heat-killed *Listeria monocytogenes* (HKLM), 10^7^ cells/mL; TLR3 agonist, poly (I:C), 1 μg/mL; TLR4 agonist, LPS from *E. coli* K-12 (LPS-EK), 10 ng/mL; TLR5 agonist, flagellin from *Salmonella enterica* serovar Typhimurium (FLA-ST), 100 ng/mL; TLR7 agonist, imiquimod, 100 ng/mL; TLR8 agonist, ssRNA40, 10 ng/mL; and TLR9 agonist, ODN2006, 500 nM. Furthermore, to find the role of TLR4 in IL-8 release, LPS-EK (10 ng/mL) or *E. coli* OMVs (10 ng/mL in total protein concentration) were treated to HMEC-1 (1 × 10^5^ cells/well) in the presence of 5% FBS at 37°C for 12 h, with or without TLR4 antagonist [LPS from *Rhodobacter sphaeroides* (InvivoGen); 1 μg/mL].

To find which signaling pathways involved in IL-8 release from endothelial cells, PBS or *E. coli* OMVs (0.5 ng/mL in total protein concentration) were treated to HMEC-1 (1 × 10^5^ cells/well) in the presence of 5% FBS at 37°C for 12 h with 0.05% dimethyl sulfoxide or signaling inhibitors (final concentration = 10 μM in 0.05% dimethyl sulfoxide). The signaling inhibitors were PD98059 (ERK1/2 inhibitor), SB203580 (p38 MAPK inhibitor), SP600125 (JNK inhibitor), LY294002 (PI3K inhibitor), and BAY11-7082 (NK-κB inhibitor), and they were obtained from Biomol Research Laboratories (Plymouth Meeting, PA, United States).

Quantification of human IL-8, human CXCL10, or mouse CXCL1 proteins in the culture supernatants or BAL fluid supernatants was performed using the DuoSet ELISA kit (R&D Systems), following the manufacturer’s instructions. The detection limit of human IL-8, human CXCL10, or mouse CXCL1 was 31.3–2,000 pg/mL, 7.8–500 pg/mL, and 15.6–1,000 pg/mL, respectively. Some samples that contained more human IL-8, human CXCL10, or mouse CXCL1 over the detection limit were diluted to measure human IL-8, human CXCL10, or mouse CXCL1 within the detection limit.

### Uptake and Internalization of OMVs by HMEC-1

PBS or *E. coli* OMVs (900 μg/mL in total protein concentration) were mixed with 1 mM of DiI fluorescent dye (Molecular Probes) and incubated at 37°C for 30 min. After adding Solution R of the ExoLutE^®^ Conditioned Medium Exosome Isolation Kit (Rosetta Exosome Inc., Seoul, South Korea), the reaction mixture was passed through a size-exclusion spun column (column S of ExoLutE^®^) to remove residual DiI. HMEC-1 cells (1 × 10^4^ cells) were seeded on 0.1% gelatin-coated cover glass overnight at 37°C and incubated with the eluents from column S (DiI-labeled OMVs, 5 μg/mL in total protein concentration; free DiI, the equivalent volume of the eluents) for 3 h at 37°C. Subsequently, the cells were washed with PBS, stained with 5-chloromethyl fluorescein diacetate (10 μM, Molecular Probes), and fixed with 4% paraformaldehyde for 30 min at room temperature. Images were acquired with a confocal microscope LSM700 (Carl Zeiss, Oberkochen, Germany).

### Western Blot

HMEC-1 cells (1 × 10^5^ cells/well) were treated with *E. coli* OMVs (0.5 ng/mL in total protein concentration) in the presence of 5% FBS at 37°C for 0, 10, 30, 60, 120, and 360 min. Whole cell lysates (20 μg in total protein amounts) were subject to SDS–PAGE and transferred to a polyvinylidene difluoride membrane. After blocking with 5% non-fat skim milk, the membrane was incubated with mouse anti-phospho-IκB (Cell Signaling Technology, Hitchin, United Kingdom), or goat anti-β-actin (Santa Cruz Biotechnology), followed by goat antimouse IgG or donkey antigoat IgG (Santa Cruz Biotechnology), which were conjugated with horseradish peroxidase. A chemiluminescent substrate (iNtRON Biotechnology Inc.) was used to visualize immunoreactive bands, which were subsequently subjected to densitometric analysis using ImageJ software (National Institute of Mental Health, Bethesda, MD, United States^1^). The density of phospho-IκB bands was normalized using that of β-actin.

### Statistical Analysis

All values were represented as means ± standard error means (SEM) with the indicated sample sizes. The sample sizes (n) represent technical and biological replicates *in vitro* studies and *in vivo* studies, respectively. *P*-values were calculated using the unpaired Student’s *t*-test as well as one- or two-way analyses of variance (ANOVA) with Bonferroni correction for multiple comparisons. A *P*-value < 0.05 was considered as statistically significant. All statistical analyses were conducted using Prism 5 software (GraphPad, La Jolla, CA, United States).

## Results

### *E. coli* OMVs Increased Neutrophil Transmigration in the Murine Lungs

From the cell culture supernatants, we isolated *E. coli* OMVs by the combination of ultrafiltration and ultracentrifugation as previously reported (Park et al., 2010; Kim et al., 2018). When we characterized isolated *E. coli* OMVs with transmission electron microscopy and dynamic light scattering, *E. coli* OMVs had spherical bilayered vesicular structures with diameters ranging from 20 to 100 nm (**Supplementary Figures 1A,B**). SDS–PAGE analysis demonstrated that OMVs contain distinct proteins when compared with whole cell lysates (**Supplementary Figure 1C**): OMVs were enriched with outer membrane proteins such as OmpA and de-enriched with cytosolic proteins such as FtsZ (**Supplementary Figure 1D**). In addition, *E. coli* OMVs harbored 75 ng of LPS per 100 ng of OMV proteins (Park et al., 2010).

**FIGURE 1:**
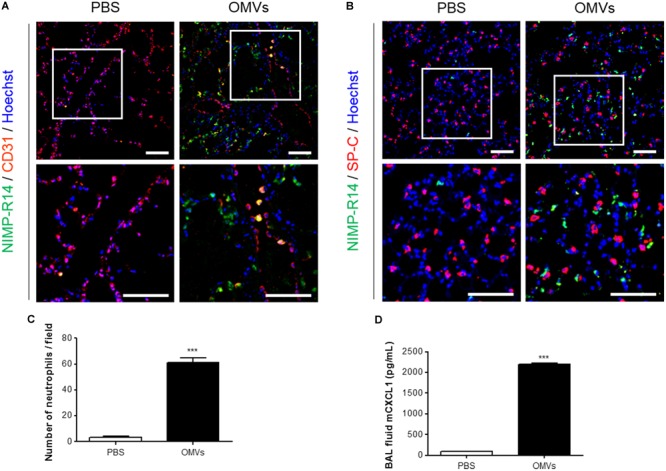
Neutrophil transmigration in murine lungs induced by *E. coli* OMVs. Wild-type mice were intraperitoneally administered with either PBS or *E. coli* OMVs (15 μg in total protein amounts per mouse). Five animals were used in each group. Different groups of mice administered with either PBS or OMVs were killed at 6 h after OMV administration, and the lung tissues as well as BAL fluids were obtained from the five mice. **(A–C)** The lung sections of five mice were immunostained with anti-NIMP-R14 (green; neutrophils) and anti-CD31 (red; endothelial cells) antibodies **(A)**, or anti-NIMP-R14 (green; neutrophils) and anti-SP-C (red; lung epithelial cells) antibodies **(B)**. The sections were then counterstained with Hoechst 33258 (blue; nuclei). Representative fluorescence images are shown here. Scale bars = 50 μm. The number of neutrophils per field was counted from five confocal microscopy images obtained from the lung sections of five mice **(C)**. **(D)** The concentration of CXCL1 was measured in the BAL fluid by ELISA (*n* = 5). Data were represented as mean ± SEM. ^∗∗∗^*P* < 0.001, calculated by unpaired Student’s *t*-test.

We first investigated the mechanisms by which *E. coli* OMVs regulate neutrophil transmigration out of the vascular spaces into the alveolar interstitial tissues, on intraperitoneal administration into mice. At 6 h after OMV administration, the majority of neutrophils were found in the alveolar interstitial tissues, whereas a minority of neutrophils were found within the vasculature (**Figure 1A**). In addition, we observed that the total number of neutrophils infiltrating the lung tissues was significantly increased in response to OMVs (**Figures 1B,C**). The concentration of the murine functional homolog of human IL-8, CXCL1, which is a neutrophil chemoattractant, was increased in the BAL fluid (**Figure 1D**). Our results demonstrated that *E. coli* OMV-induced neutrophil transmigration in the lungs was associated with the increased release of CXCL1 in the BAL fluid as a consequence of treatment with OMVs.

### Endothelial Cells Were Main Functional Targets of OMVs

**FIGURE 2:**
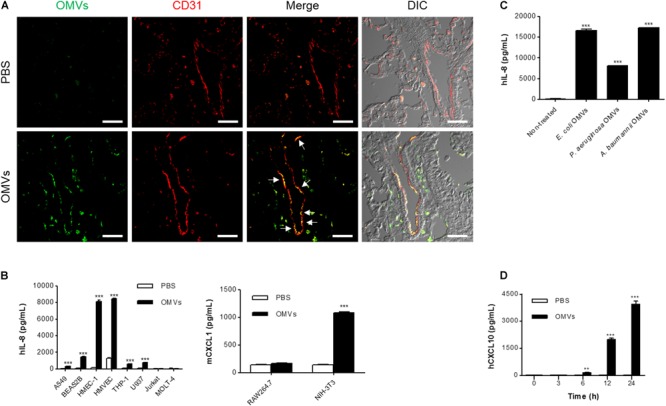
Endothelial cells as main functional targets of OMVs. **(A)** Wild-type mice were intraperitoneally administered with either PBS or *E. coli* OMVs (15 μg in total protein amount per mouse). Five animals were used in each group. At 3 h after OMV administration, the lung tissues were retrieved. The lung sections of five mice were immunostained with anti-OMVs (green; *E. coli* OMVs) and anti-CD31 (red; endothelial cells) antibodies. Representative fluorescence images are shown here. Scale bars = 30 μm. White arrows indicate *E. coli* OMV-positive endothelial cells. **(B)** After treating *E. coli* OMVs (0.5 ng/mL in total protein concentration) to various human (A549, BEAS2B, HMEC-1, HMVEC, THP-1, U937, Jurkat, and MOLT-4) and mouse cells (RAW264.7 and NIH-3T3), the concentrations of human IL-8 or mouse CXCL1 were measured in the culture supernatants by ELISA (*n* = 3). **(C)** After treating various OMVs (*E. coli, P. aeruginosa*, and *A. baumannii* OMVs; 0.5 ng/mL in total protein concentration) to human HMEC-1 endothelial cells, the concentration of human IL-8 was measured in the culture supernatants by ELISA (*n* = 3). **(D)** After treating *E. coli* OMVs (0.5 ng/mL in total protein concentration) to human HMEC-1 endothelial cells, the concentrations of human CXCL10 were measured in the culture supernatants by ELISA (*n* = 3). Data were represented as mean ± SEM. ^∗∗^*P* < 0.01 and ^∗∗∗^*P* < 0.001, respectively, calculated by two-way **(B,D)** or one-way ANOVA **(C)** with Bonferroni correction for multiple comparisons.

We next attempted to identify the main functional target cells of *E. coli* OMVs. At 3 h after OMV administration, *E. coli* OMV components were colocalized extensively with CD31-positive endothelial cells (**Figure 2A**), suggesting that endothelial cells could be the cell targets that avidly uptake OMVs in the circulation. This result also suggests that OMVs might migrate to the lung tissues via the blood vessels by functional interaction with endothelial cells. Next, we treated various doses of *E. coli* OMVs to diverse cell types: epithelial cells (A549 and BEAS2B), endothelial cells (HMEC-1 and HMVEC), monocytes (THP-1 and U937), T cells (Jurkat and MOLT-4), macrophages (RAW264.7), and fibroblasts (NIH-3T3). We measured human IL-8 from the culture supernatants of human A549, BEAS2B, HMEC-1, HMVEC, THP-1, U937, Jurkat, and MOLT-4 cells, and mouse CXCL1 (a murine functional homolog of human IL-8; Mohsenin et al., 2007) from those of mouse RAW264.7 and NIH-3T3 cells. Although the concentrations of human IL-8 or mouse CXCL1 were significantly increased in the culture supernatants of several cell types in dose-dependent manners, human endothelial cells were most prominent among them (**Supplementary Figure 2**). Indeed, when the several types of cells were exposed to the same concentration of *E. coli* OMVs (0.5 ng/mL in total protein concentration), the concentrations of IL-8 reached extremely high levels in endothelial cells (**Figure 2B**). In addition, IL-8 release from endothelial cells was also induced by OMVs from other Gram-negative bacteria, such as *P. aeruginosa* and *A. baumannii* (**Figure 2C**). We also found that the concentration of human CXCL10 was also increased in the conditioned media of HMEC-1 exposed to *E. coli* OMVs (0.5 ng/mL in total protein concentration) in a time-dependent manner (**Figure 2D**). Taken together, these results suggest that endothelial cells might be functional targets of OMVs in the lung tissue, and respond to OMVs by releasing IL-8.

### TLR4 and NF-κB Were Involved in IL-8 Release From OMV-Stimulated Endothelial Cells

**FIGURE 3:**
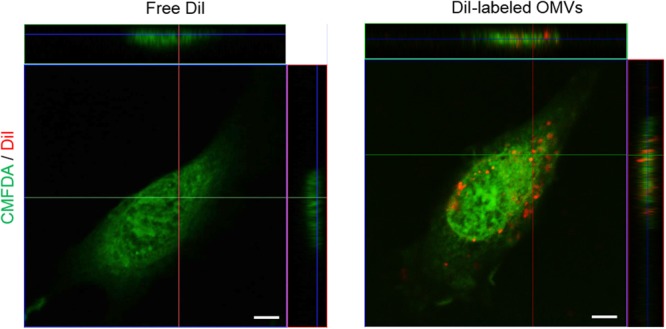
Internalization of OMVs into endothelial cells. Uptake and internalization of free DiI control or DiI-labeled *E. coli* OMVs (red fluorescent signal) by HMEC-1 labeled with 5-chloromethylfluorescein (CMFDA, green fluorescent signal) was examined using a confocal microscopy. Representative three-dimensional fluorescence images are shown here. Scale bars = 5 μm. Note that free DiI control or DiI-labeled *E. coli* OMVs were prepared by a size-exclusion spun column to remove residual DiI: free DiI control itself does not contain any fluorescent signal. Low magnificent two-dimensional fluorescence images and high magnificent two-dimensional fluorescence images with representative three-dimensional fluorescence images are shown in **Supplementary Figure 3**.

**FIGURE 4:**
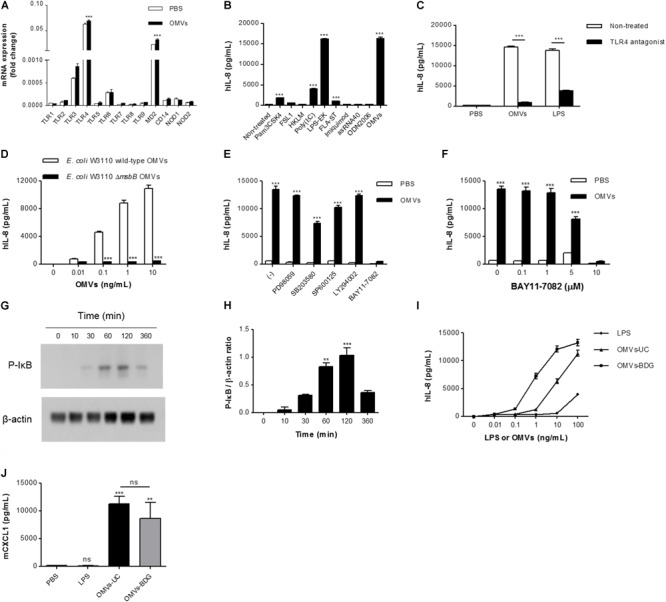
The roles of TLR4 and NF-κB in IL-8 release from OMV-stimulated endothelial cells. **(A)** Total RNA was isolated from HMEC-1 treated with PBS or *E. coli* OMVs (0.5 ng/mL) for 12 h, and mRNA expression of TLRs (TLR1–TLR9), MD2, CD14, NOD1, and NOD2 were analyzed by real-time RT-PCR (*n* = 3). Fold changes were calculated by dividing the expression of each gene by that of GAPDH. **(B)** HMEC-1 was treated with *E. coli* OMVs (0.5 ng/mL in total protein concentration) or TLR agonists for 12 h, and the concentrations of IL-8 were quantified in the culture supernatants by ELISA (*n* = 3). Human TLR agonists were used as follows: TLR1/2 agonist, Pam_3_CSK_4_, 100 ng/mL; TLR2/6 agonist, FSL1, 100 ng/mL; TLR2 agonist, HKLM, 10^7^ cells/mL; TLR3 agonist, poly (I:C), 1 μg/mL; TLR4 agonist, LPS-EK, 10 ng/mL; TLR5 agonist, FLA-ST, 100 ng/mL; TLR7 agonist, imiquimod, 100 ng/mL; TLR8 agonist, ssRNA40, 10 ng/mL; and TLR9 agonist, ODN2006, 500 nM. **(C)** PBS, *E. coli* OMVs (10 ng/mL in total protein concentration), or LPS-EK (10 ng/mL) were treated to HMEC-1, with or without TLR4 antagonist (1 μg/mL) for 12 h, and the concentrations of IL-8 were measured in the culture supernatants by ELISA (*n* = 3). **(D)** Various concentrations (0, 0.01, 0.1, 1, and 10 ng/mL in total protein concentrations) of *E. coli* W3110 wild-type OMVs or *E. coli* W3110 *ΔmsbB* mutant OMVs were treated to HMEC-1 for 12 h, and the concentrations of IL-8 were measured in the culture supernatants by ELISA. **(E)** PBS or *E. coli* OMVs (0.5 ng/mL in total protein concentration) were treated to HMEC-1 for 12 h with 0.05% dimethyl sulfoxide (–) or the following signaling inhibitors (final concentration = 10 μM in 0.05% dimethyl sulfoxide): PD98059 (ERK1/2 inhibitor), SB203580 (p38 MAPK inhibitor), SP600125 (JNK inhibitor), LY294002 (PI3K inhibitor), and BAY11-7082 (NK-κB inhibitor), and the concentrations of IL-8 were measured in the culture supernatants by ELISA (*n* = 3). **(F)** PBS or *E. coli* OMVs (0.5 ng/mL in total protein concentration) were treated to HMEC-1 for 12 h with various concentrations of BAY11-7082 (0, 0.1, 1, 5, and 10 μM), and the concentrations of IL-8 were measured in the culture supernatants by ELISA (*n* = 3). **(G,H)** HMEC-1 were treated with PBS or *E. coli* OMVs (0.5 ng/mL in total protein concentration) for 0, 10, 30, 60, 120, or 360 min. Whole cell lysates (20 μg in total protein amount) were subjected to analyzing the expression of phosphorylated-IκB (P-IκB) and β-actin by Western blot. The representative blot of two independent experiments **(G)** and the average values of the relative ratios calculated by dividing the densitometry quantification values for P-IκB by those of β-actin **(H)**. **(I)** Various concentrations (0, 0.01, 0.1, 1, 10, and 100 ng/mL) of *E. coli* LPS, OMVs-UC, or OMVs-BDG were treated to HMEC-1 for 12 h, and the concentrations of IL-8 were measured in the culture supernatants by ELISA. LPS, LPS isolated from *E. coli*; OMVs-UC, OMVs isolated by the combination of ultrafiltration and ultracentrifugation (UC); OMVs-BDG, OMVs isolated by the combination of ultrafiltration, ultracentrifugation, buoyant density gradient ultracentrifugation (BDG), and ultracentrifugation. **(J)** Wild-type mice were intraperitoneally administered with PBS, *E. coli* LPS (11.25 μg per mouse), or *E. coli* OMVs-UC (15 μg in total protein amounts per mouse) or *E. coli* OMVs-BDG (15 μg in total protein amounts per mouse). Five mice were used for each group. At 6 h after administration, the mice were killed. The BAL fluids were retrieved, and the concentration of CXCL1 was measured in the BAL fluid by ELISA (*n* = 5). Data were represented as mean ± SEM. ns, non-significant; ^∗∗^*P* < 0.01; ^∗∗∗^*P* < 0.001, calculated by one-way **(B,H,J)** or two-way ANOVA **(A,C–F)** with Bonferroni correction for multiple comparisons.

We next investigated the mechanisms underlying IL-8 release from endothelial cells. When HMEC-1 were incubated with DiI-labeled *E. coli* OMVs, we found strong red fluorescent signals indicating DiI in the cytoplasm of HMEC-1 (**Figure 3** and **Supplementary Figure 3**). These results indicate that HMEC-1 uptake and internalize *E. coli* OMVs. Because endothelial cells were known to express TLRs, particularly TLR4, which recognizes LPS on Gram-negative bacteria and OMVs (Faure et al., 2000), we measured the mRNA expression of TLRs, MD2 [a coreceptor cooperating with TLR4 to bind LPS (Shimazu et al., 1999)], and CD14 in HMEC-1. Real-time RT-PCR revealed that TLR4 and MD2 were expressed at higher levels than other TLRs, CD14, and cytosolic NOD receptors (NOD1 and NOD2) in HMEC-1 (**Figure 4A**). Furthermore, we observed that the expression of TLR4 and MD2 was a little upregulated in HMEC-1 at 12 h after exposed to 0.5 ng/mL of *E. coli* OMVs (**Figure 4A**). Although expression levels of NOD1 and NOD2 in HMEC-1 are extremely low (**Figure 4A**), OMVs derived from Gram-negative bacteria such as *Helicobacter pylori, P. aeruginosa*, and *Neisseria gonorrhoeae* activate NF-κB of intestinal epithelial cells in an NOD1-dependent manner (Kaparakis et al., 2010). Thus, further studies to elucidate endothelial NOD1/NOD2 pathways in OMV-mediated neutrophil recruitment in the lung would be valuable. To characterize TLR functions in HMEC-1, we treated various TLR agonists to HMEC-1 and observed that IL-8 release was most significantly increased by treating with LPS-EK, a TLR4 agonist (**Figure 4B**). This result was corroborated by the suppression of IL-8 release by *E. coli* OMVs or LPS-EK in the presence of a TLR4 antagonist (**Figure 4C**). In addition, we observed that *E. coli ΔmsbB* mutant OMV-treated HMEC-1 did not produce significant amounts of IL-8, whereas high levels of IL-8 were produced by *E. coli* wild-type OMV-treated cells (**Figure 4D**). As *E. coli ΔmsbB* mutant has impaired lipid A structure due to the deletion of gene encoding lipid A acyltransferase (*msbB*), *E. coli ΔmsbB* mutant OMVs cannot stimulate TLR4 (Somerville et al., 1999; Kim et al., 2009, 2017). These results lead us to the conclusion that *E. coli* OMVs induce IL-8 release from endothelial cells via TLR4.

We further investigated to determine which signaling pathways are associated with *E. coli* OMV-induced IL-8 release from endothelial cells, using inhibitors of various signaling pathways (**Figure 4E**). PD98059 (ERK1/2 inhibitor) and LY294002 (PI3K inhibitor) did not suppress OMV-induced release of IL-8. SB203580 (p38 MAPK inhibitor) and SP600125 (JNK inhibitor) slightly attenuated IL-8 release, whereas BAY11-7082 (NK-κB inhibitor) completely abrogated OMV-induced IL-8 release (**Figure 4E**). BAY11-7082 dose-dependently inhibited IL-8 release (**Figure 4F**). Because phosphorylation of the NF-κB inhibitor IκB is a prerequisite for IκB degradation and consequent NF-κB activation (Karin and Ben-Neriah, 2000; Shimada and Rajagopalan, 2010), we treated HMEC-1 with *E. coli* OMVs and found that *E. coli* OMVs significantly increased IκB phosphorylation within an hour (**Figures 4G,H**). These results indicate that NF-κB is involved in propagating TLR4 signaling pathways toward IL-8 release from OMV-stimulated endothelial cells.

To exclude the possibility that our *in vitro* and *in vivo* observed biological activities of OMVs isolated by the combination of ultrafiltration and ultracentrifugation are derived from non-vesicular potential contaminants such as flagella, fimbria, pili, LPS, and large protein complexes/aggregates (Kulp and Kuehn, 2010; Klimentová and Stulík, 2015), we further isolated OMVs by the combination of ultrafiltration, ultracentrifugation, buoyant density gradient ultracentrifugation, and ultracentrifugation (OMVs-BDG), as previously reported (Kim et al., 2017). Similarly with *E. coli* OMVs isolated by the combination of ultrafiltration and ultracentrifugation (OMVs-UC; **Supplementary Figure 1**), *E. coli* OMVs-BDG had spherical bilayered vesicular structures with diameters ranging from 20 to 100 nm (**Supplementary Figures 4A,B**). In addition, *E. coli* OMVs-BDG were enriched with outer membrane proteins such as OmpA and de-enriched with cytosolic proteins such as FtsZ (**Supplementary Figure 4C**). However, *E. coli* OMVs-BDG harbored 102 ng of LPS per 100 ng of OMV proteins, whereas 100 ng of *E. coli* OMVs-UC harbored 75 ng of LPS. *In vitro* experiments showed that *E. coli* OMVs-BDG were more potent than *E. coli* OMVs-UC in inducing IL-8 release from HMEC-1 (**Figure 4I**). On intraperitoneal administration into a mouse, *E. coli* OMVs-UC were slightly more potent than *E. coli* OMVs-BDG in inducing CXCL1 from the BAL fluid (**Figure 4J**). Furthermore, non-vesicular free LPS isolated from *E. coli* was much less potent than both *E. coli* OMVs-UC and OMVs-BDG (**Figures 4I,J**). Taken together, all these observations suggested that our observed biological activities of OMVs isolated by the combination of ultrafiltration and ultracentrifugation are not mainly derived from the non-vesicular potential contaminants, especially non-vesicular free LPS.

### OMV-Induced Neutrophil Transmigration in the Murine Lungs Was Suppressed in TLR4 Knockout Mice

**FIGURE 5:**
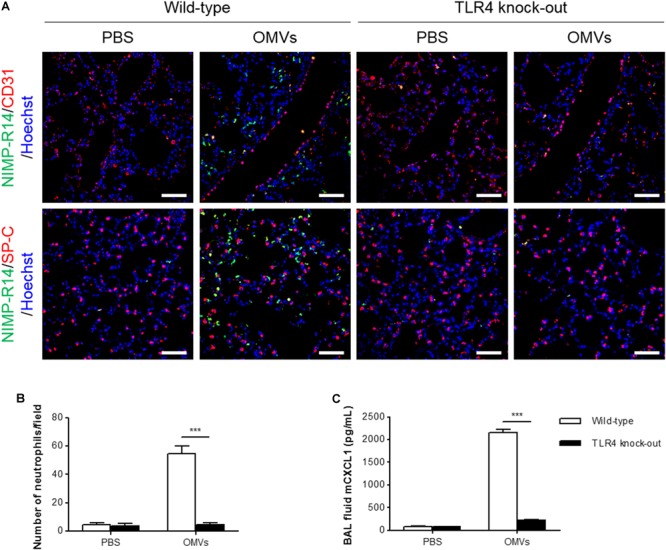
Suppression of OMV-induced neutrophil transmigration in murine lungs of TLR4 knockout mice. Wild-type and TLR4 knockout mice were intraperitoneally administered with PBS or *E. coli* OMVs (15 μg in total protein amount) for 6 h. Five animals were used in each group. **(A,B)** The lung sections of five mice at 6 h after *E. coli* OMV introduction were immunostained with anti-NIMP-R14 (green; neutrophils) and anti-CD31 (red; endothelial cells) or anti-SP-C (red; lung epithelial cells) antibodies, and counterstained with Hoechst 33258 (blue; nuclei). Representative fluorescence images are shown here. Scale bars = 50 μm. The number of neutrophils per field was counted from five confocal microscopy images obtained from the lung sections of five mice **(B)**. **(C)** The concentration of CXCL1 was measured in the BAL fluid by ELISA (*n* = 5). Data were represented as mean ± SEM. ^∗∗∗^*P* < 0.001, calculated by two-way ANOVA with Bonferroni correction for multiple comparisons.

*Escherichia coli* OMVs induced IL-8 release from endothelial cells in a TLR4-dependent manner *in vitro*, and IL-8 can induce neutrophil transendothelial migration (Smith et al., 1991). Thus, we determined whether OMV-induced neutrophil transmigration in the lungs was impaired in TLR4 knockout mice. We observed that neutrophils were mostly located in the alveolar interstitial tissues of wild-type mice, whereas few if any neutrophils were found in the same tissues of TLR4 knockout mice (**Figure 5A**). In addition, we found that on OMV administration, the number of neutrophils infiltrating the lung tissues was significantly decreased in TLR4 knockout versus wild-type mice (**Figures 5A,B**). The concentration of CXCL1 in the BAL fluid was also significantly decreased in TLR4 knockout mice, when compared with wild-type mice (**Figure 5C**). Our results demonstrated that TLR4 is important in OMV-induced neutrophil transmigration in the lungs and the release of CXCL1 in the BAL fluid.

## Discussion

We have previously demonstrated that *E. coli* OMVs induce neutrophilic inflammation in the lungs (Park et al., 2010; Jang et al., 2015) and upregulate the expression of cell adhesion molecules in the lung endothelium (Kim J.H. et al., 2013). However, the molecular mechanisms by which OMVs recruit neutrophils and allow them to adhere and transmigrate into the lung tissues are not well understood. In this study, we demonstrated that *E. coli* OMVs induce expression of the neutrophil chemoattractant CXCL1, a murine functional homolog of human IL-8, and transmigration of neutrophils into the lung tissues *in vivo*. *E. coli* OMVs significantly increased IL-8 release from human endothelial cells *in vitro* in a TLR4- and NK-κB-dependent manner. Finally, CXCL1 induction and the transmigration of neutrophils into the lung tissues were remarkably diminished in TLR4 knockout mice.

Circulating neutrophils migrate into the lung tissues during bacterium-induced acute lung injury and mediate the lung damage (Martin, 2002; Craig et al., 2009). The lung damage can be reduced by depleting neutrophils or blocking neutrophil chemoattractants, suggesting that neutrophil trafficking into the lungs is crucial in acute lung injury (Craig et al., 2009). Neutrophil chemoattractants are produced by resident cells, such as endothelial cells, epithelial cells, and alveolar macrophages, as well as infiltrating leukocytes (Huber et al., 1991; Middleton et al., 1997). In the lung tissues, the source of IL-8 depends on the types of cells interacting with bacteria or with their components for the first time. For instance, during lower respiratory tract bacterial infection, alveolar epithelial cells and macrophages release chemoattractants for neutrophils, on interacting with bacteria (Mizgerd and Skerrett, 2008). Similarly, *P. aeruginosa* OMVs can activate alveolar macrophages after intranasal administration into mice and subsequently induce expression of CXCL1 and infiltration of neutrophils into the lung tissues (Park et al., 2013). Unlike pulmonary inflammation, endothelial cells are the first cells to encounter bacteria in sepsis (Peters et al., 2003). In the *E. coli* OMV-induced SIRS model, a significant amount of OMVs were detected in the blood and lungs at 3 h after intraperitoneal administration of OMVs (Jang et al., 2015). In this study, we showed that *E. coli* OMVs were colocalized with CD31-positive lung endothelial cells on intraperitoneal administration. Subsequently, OMV-stimulated endothelial cells produce CXCL1, allowing circulating neutrophils to adhere and transmigrate across the endothelium toward an increasing gradient of CXCL1. Additional *in vitro* experiments showed that *E. coli* OMVs were taken up and internalized by HMEC-1.

Outer membrane vesicles are enriched with a variety of pathogen-associated molecular patterns, including lipoproteins, LPS, flagellin, and CpG DNA, most of which are TLR ligands (Ellis et al., 2010). These TLR ligands are recognized by diverse sentinel cells, triggering the rapid release of cytokines and chemokines, paralleled by increased expression of cell adhesion molecules (Akira and Takeda, 2004; Meylan et al., 2006). In particular, these cytokines and chemokines are necessary for the transmigration of circulating leukocytes to the infection sites and for the removal of pathogens (Agace et al., 1995; Aird, 2003). In addition, upregulation of cell adhesion molecules, such as intercellular adhesion molecule-1 (ICAM-1) and E-selectin, enhances the adhesion of leukocytes to the endothelium (Haraldsen et al., 1996; Ley et al., 2007). OMVs derived from *Porphyromonas gingivalis* or *E. coli* increased the expression of ICAM-1 and E-selectin, resulting in neutrophil infiltration into the inflamed tissues (Srisatjaluk et al., 1999; Kim J.H. et al., 2013). Moreover, OMVs triggered the release of TNF-α, IL-6, IL-1β, and IL-8 from epithelial cells, macrophages, and dendritic cells (Ismail et al., 2003; Bauman and Kuehn, 2006; Alaniz et al., 2007; Ellis et al., 2010). However, only a few studies have investigated the functional roles of OMV-induced cytokines/chemokines, and as far as we know, there are no reports regarding OMV-induced release of IL-8 from endothelial cells. In this study, we demonstrated that *E. coli* OMVs increased the release of IL-8/CXCL1 from endothelial cells, which were responsible for neutrophil transmigration. It has been shown that *E. coli* can induce IL-8 expression in endothelial cells (Galanakis et al., 2006) and IL-8 can induce neutrophil transendothelial migration (Smith et al., 1991). Thus, as observed with *E. coli* OMVs, we can speculate that live *E. coli* can recruit neutrophils into the lungs by stimulation of endothelial CXCL1 release. Future studies to determine the physiological concentration of OMVs during infections may help us to further understand the role of OMVs and their relations with bacteria in clinical diseases (Park et al., 2013).

Unlike other cells, endothelial cells predominantly express TLR4, which can activate both MyD88-dependent and -independent pathways (Andonegui et al., 2003, 2009). After binding of LPS, TLR4 stimulates the early and late activation of NF-κB, resulting in the production of TNF-α, IL-8, CXCL1, and ICAM-1 (Dauphinee and Karsan, 2006; Kagan et al., 2008; Gais et al., 2012). In addition, endothelial TLR4 can stimulate other signaling pathways, such as ERK1/2, p38 MAPK, and JNK, to produce cytokines and chemokines (Dauphinee and Karsan, 2006). In this study, *E. coli* OMVs induced IL-8 release, predominantly by NF-κB activation. Moreover, TLR4 inhibition completely blocked IL-8 release in OMV-stimulated human endothelial cells and the release of CXCL1 in the BAL fluid of mice treated with *E. coli* OMVs. Therefore, we showed the importance of OMV-associated LPS and TLR4 on endothelial cells in OMV-induced production of neutrophil chemoattractants, although it is possible that internalized OMVs can also activate non-canonical inflammasomes and cytokine release via host guanylate-binding proteins in a TLR4-independent manner (Santos et al., 2018).

In summary, our study revealed that *E. coli* OMVs potently stimulate expression of IL-8/CXCL1 in the lung endothelium via TLR4- and NF-κB-dependent pathways, resulting in neutrophil transmigration into the inflamed tissues. Our findings will improve the understanding of the proinflammatory activities of OMVs in vascular inflammation by bacterial infection.

## Author Contributions

JL, YJY, JHK, T-YR, DDV, and YSG conceived and designed the research. JL, YJY, JHK, NTHD, GG, ST, K-SP, HTP, and CL performed the experiments. JL, YJY, JHK, and NTHD analyzed the data. JL, YJY, JHK, ST, T-YR, DDV, and YSG wrote the manuscript with editing and input from all authors.

## Conflict of Interest Statement

The authors declare that the research was conducted in the absence of any commercial or financial relationships that could be construed as a potential conflict of interest.

## Footnotes

1 http://rsb.info.nih.gov/ij

## References

[B1] Agace W. W. Patarroyo M. Svensson M. Carlemalm E. Svanborg C. (1995). *Escherichia coli* induces transuroepithelial neutrophil migration by an intercellular adhesion molecule-1-dependent mechanism. *Infect. Immun.* 63 4054–4062. 7558319 10.1128/iai.63.10.4054-4062.1995 PMC173570

[B2] Aird W. C. (2003). The role of the endothelium in severe sepsis and multiple organ dysfunction syndrome. *Blood* 101 3765–3777. 10.1182/blood-2002-06-1887 12543869

[B3] Akira S. Takeda K. (2004). Toll-like receptor signalling. *Nat. Rev. Immunol.* 4 499–511. 10.1038/nri1391 15229469

[B4] Alaniz R. C. Deatherage B. L. Lara J. C. Cookson B. T. (2007). Membrane vesicles are immunogenic facsimiles of *Salmonella* typhimurium that potently activate dendritic cells, prime B and T cell responses, and stimulate protective immunity in vivo. *J. Immunol.* 179 7692–7701. 10.4049/jimmunol.179.11.7692 18025215

[B5] Andonegui G. Bonder C. S. Green F. Mullaly S. C. Zbytnuik L. Raharjo E. (2003). Endothelium-derived Toll-like receptor-4 is the key molecule in LPS-induced neutrophil sequestration into lungs. *J. Clin. Invest.* 111 1011–1020. 10.1172/JCI16510 12671050 PMC152584

[B6] Andonegui G. Zhou H. Bullard D. Kelly M. M. Mullaly S. C. Mcdonald B. (2009). Mice that exclusively express TLR4 on endothelial cells can efficiently clear a lethal systemic Gram-negative bacterial infection. *J. Clin. Invest.* 119 1921–1930. 19603547 10.1172/JCI36411 PMC2701859

[B7] Annane D. Bellissant E. Cavaillon J. M. (2005). Septic shock. *Lancet* 365 63–78. 10.1016/S0140-6736(04)17667-8 15639681

[B8] Bauman S. J. Kuehn M. J. (2006). Purification of outer membrane vesicles from *Pseudomonas aeruginosa* and their activation of an IL-8 response. *Microbes Infect.* 8 2400–2408. 10.1016/j.micinf.2006.05.001 16807039 PMC3525494

[B9] Beveridge T. J. Kadurugamuwa J. L. (1996). Periplasm, periplasmic spaces, and their relation to bacterial wall structure: novel secretion of selected periplasmic proteins from *Pseudomonas aeruginosa*. *Microb. Drug Resist.* 2 1–8. 10.1089/mdr.1996.2.1 9158716

[B10] Choi D. S. Kim D. K. Choi S. J. Lee J. Choi J. P. Rho S. (2011). Proteomic analysis of outer membrane vesicles derived from *Pseudomonas aeruginosa*. *Proteomics* 11 3424–3429. 10.1002/pmic.201000212 21751344

[B11] Costerton J. W. Ingram J. M. Cheng K. J. (1974). Structure and function of the cell envelope of Gram-negative bacteria. *Bacteriol. Rev.* 38 87–110. 4601163 10.1128/br.38.1.87-110.1974 PMC413842

[B12] Craig A. Mai J. Cai S. Jeyaseelan S. (2009). Neutrophil recruitment to the lungs during bacterial pneumonia. *Infect. Immun.* 77 568–575. 10.1128/IAI.00832-08 19015252 PMC2632043

[B13] Dauphinee S. M. Karsan A. (2006). Lipopolysaccharide signaling in endothelial cells. *Lab. Invest.* 86 9–22. 10.1038/labinvest.3700366 16357866

[B14] Ellis T. N. Leiman S. A. Kuehn M. J. (2010). Naturally produced outer membrane vesicles from *Pseudomonas aeruginosa* elicit a potent innate immune response via combined sensing of both lipopolysaccharide and protein components. *Infect. Immun.* 78 3822–3831. 10.1128/IAI.00433-10 20605984 PMC2937433

[B15] Faure E. Equils O. Sieling P. A. Thomas L. Zhang F. X. Kirschning C. J. (2000). Bacterial lipopolysaccharide activates NF-kappaB through Toll-like receptor 4 (TLR-4) in cultured human dermal endothelial cells. Differential expression of TLR-4 and TLR-2 in endothelial cells. *J. Biol. Chem.* 275 11058–11063. 10.1074/jbc.275.15.11058 10753909

[B16] Gais P. Reim D. Jusek G. Rossmann-Bloeck T. Weighardt H. Pfeffer K. (2012). Cutting edge: divergent cell-specific functions of MyD88 for inflammatory responses and organ injury in septic peritonitis. *J. Immunol.* 188 5833–5837. 10.4049/jimmunol.1200038 22586041

[B17] Galanakis E. Di Cello F. Paul-Satyaseela M. Kim K. S. (2006). *Escherichia coli* K1 induces IL-8 expression in human brain microvascular endothelial cells. *Eur. Cytokine Netw.* 17 260–265. 17353159

[B18] Haraldsen G. Kvale D. Lien B. Farstad I. N. Brandtzaeg P. (1996). Cytokine-regulated expression of E-selectin, intercellular adhesion molecule-1 (ICAM-1), and vascular cell adhesion molecule-1 (VCAM-1) in human microvascular endothelial cells. *J. Immunol.* 156 2558–2565. 8786319

[B19] Harari O. A. Alcaide P. Ahl D. Luscinskas F. W. Liao J. K. (2006). Absence of TRAM restricts Toll-like receptor 4 signaling in vascular endothelial cells to the MyD88 pathway. *Circ. Res.* 98 1134–1140. 10.1161/01.RES.0000220105.85182.28 16574902 PMC2701732

[B20] Hol J. Wilhelmsen L. Haraldsen G. (2010). The murine IL-8 homologues KC, MIP-2, and LIX are found in endothelial cytoplasmic granules but not in Weibel-Palade bodies. *J. Leukoc. Biol.* 87 501–508. 10.1189/jlb.0809532 20007247

[B21] Horstman A. L. Kuehn M. J. (2002). Bacterial surface association of heat-labile enterotoxin through lipopolysaccharide after secretion via the general secretory pathway. *J. Biol. Chem.* 277 32538–32545. 10.1074/jbc.M203740200 12087095 PMC4391702

[B22] Huber A. R. Kunkel S. L. Todd R. F. III Weiss S. J. (1991). Regulation of transendothelial neutrophil migration by endogenous interleukin-8. *Science* 254 99–102. 10.1126/science.1718038 1718038

[B23] Ismail S. Hampton M. B. Keenan J. I. (2003). *Helicobacter pylori* outer membrane vesicles modulate proliferation and interleukin-8 production by gastric epithelial cells. *Infect. Immun.* 71 5670–5675. 10.1128/IAI.71.10.5670-5675.2003 14500487 PMC201067

[B24] Jang S. C. Kim S. R. Yoon Y. J. Park K. S. Kim J. H. Lee J. (2015). In vivo kinetic biodistribution of nano-sized outer membrane vesicles derived from bacteria. *Small* 11 456–461. 10.1002/smll.201401803 25196673

[B25] Kagan J. C. Su T. Horng T. Chow A. Akira S. Medzhitov R. (2008). TRAM couples endocytosis of Toll-like receptor 4 to the induction of interferon-beta. *Nat. Immunol.* 9 361–368. 10.1038/ni1569 18297073 PMC4112825

[B26] Kaparakis M. Turnbull L. Carneiro L. Firth S. Coleman H. A. Parkington H. C. (2010). Bacterial membrane vesicles deliver peptidoglycan to NOD1 in epithelial cells. *Cell. Microbiol.* 12 372–385. 10.1111/j.1462-5822.2009.01404.x 19888989

[B27] Karin M. Ben-Neriah Y. (2000). Phosphorylation meets ubiquitination: the control of NF-[kappa]B activity. *Annu. Rev. Immunol.* 18 621–663. 10.1146/annurev.immunol.18.1.621 10837071

[B28] Kim C. W. Lee H. M. Lee T. H. Kang C. Kleinman H. K. Gho Y. S. (2002). Extracellular membrane vesicles from tumor cells promote angiogenesis via sphingomyelin. *Cancer Res.* 62 6312–6317. 12414662

[B29] Kim J. H. Lee J. Park K. S. Hong S. W. Gho Y. S. (2018). Drug repositioning to alleviate systemic inflammatory response syndrome caused by Gram-negative bacterial outer membrane vesicles. *Adv. Healthc. Mater.* 7:e1701476. 10.1002/adhm.201701476 29683274

[B30] Kim J. H. Yoon Y. J. Lee J. Choi E. J. Yi N. Park K. S. (2013). Outer membrane vesicles derived from *Escherichia coli* up-regulate expression of endothelial cell adhesion molecules in vitro and in vivo. *PLoS One* 8:e59276. 10.1371/journal.pone.0059276 23516621 PMC3597602

[B31] Kim O. Y. Hong B. S. Park K. S. Yoon Y. J. Choi S. J. Lee W. H. (2013). Immunization with *Escherichia coli* outer membrane vesicles protects bacteria-induced lethality via Th1 and Th17 cell responses. *J. Immunol.* 190 4092–4102. 10.4049/jimmunol.1200742 23514742

[B32] Kim O. Y. Park H. T. Dinh N. T. H. Choi S. J. Lee J. Kim J. H. (2017). Bacterial outer membrane vesicles suppress tumor by interferon-γ-mediated antitumor response. *Nat. Commun.* 8:626. 10.1038/s41467-017-00729-8 28931823 PMC5606984

[B33] Kim S. H. Kim K. S. Lee S. R. Kim E. Kim M. S. Lee E. Y. (2009). Structural modifications of outer membrane vesicles to refine them as vaccine delivery vehicles. *Biochim. Biophys. Acta* 1788 2150–2159. 10.1016/j.bbamem.2009.08.001 19695218 PMC5007125

[B34] Klimentová J. Stulík J. (2015). Methods of isolation and purification of outer membrane vesicles from Gram-negative bacteria. *Microbiol. Res.* 170 1–9. 10.1016/j.micres.2014.09.006 25458555

[B35] Kuehn M. J. Kesty N. C. (2005). Bacterial outer membrane vesicles and the host-pathogen interaction. *Genes Dev.* 19 2645–2655. 10.1101/gad.1299905 16291643

[B36] Kulp A. Kuehn M. J. (2010). Biological functions and biogenesis of secreted bacterial outer membrane vesicles. *Annu. Rev. Microbiol.* 64 163–184. 10.1146/annurev.micro.091208.073413 20825345 PMC3525469

[B37] Kwon S. O. Gho Y. S. Lee J. C. Kim S. I. (2009). Proteome analysis of outer membrane vesicles from a clinical *Acinetobacter baumannii* isolate. *FEMS Microbiol. Lett.* 297 150–156. 10.1111/j.1574-6968.2009.01669.x 19548894

[B38] Lee C. H. Tsai C. M. (1999). Quantification of bacterial lipopolysaccharides by the purpald assay: measuring formaldehyde generated from 2-keto-3-deoxyoctonate and heptose at the inner core by periodate oxidation. *Anal. Chem.* 267 161–168. 9918668 10.1006/abio.1998.2961

[B39] Lee E. Y. Bang J. Y. Park G. W. Choi D. S. Kang J. S. Kim H. J. (2007). Global proteomic profiling of native outer membrane vesicles derived from *Escherichia coli*. *Proteomics* 7 3143–3153. 10.1002/pmic.200700196 17787032

[B40] Lee J. Kim O. Y. Gho Y. S. (2016). Proteomic profiling of Gram-negative bacterial outer membrane vesicles: current perspectives. *Proteomics Clin. Appl.* 10 897–909. 10.1002/prca.201600032 27480505

[B41] Ley K. Laudanna C. Cybulsky M. I. Nourshargh S. (2007). Getting to the site of inflammation: the leukocyte adhesion cascade updated. *Nat. Rev. Immunol.* 7 678–689. 10.1038/nri2156 17717539

[B42] Livak K. J. Schmittgen T. D. (2001). Analysis of relative gene expression data using real-time quantitative PCR and the Method. *Methods* 25 402–408. 10.1006/meth.2001.1262 11846609

[B43] Martin T. R. (2002). Neutrophils and lung injury: getting it right. *J. Clin. Invest.* 110 1603–1605. 10.1172/JCI0217302 12464663 PMC151642

[B44] Meylan E. Tschopp J. Karin M. (2006). Intracellular pattern recognition receptors in the host response. *Nature* 442 39–44. 10.1038/nature04946 16823444

[B45] Middleton J. Neil S. Wintle J. Clark-Lewis I. Moore H. Lam C. (1997). Transcytosis and surface presentation of IL-8 by venular endothelial cells. *Cell* 91 385–395. 10.1016/S0092-8674(00)80422-5 9363947

[B46] Mizgerd J. P. Skerrett S. J. (2008). Animal models of human pneumonia. *Am. J. Physiol. Lung Cell. Mol. Physiol.* 294 L387–L398. 10.1152/ajplung.00330.2007 18162603

[B47] Mohsenin A. Burdick M. D. Molina J. G. Keane M. P. Blackburn M. R. (2007). Enhanced CXCL1 production and angiogenesis in adenosine-mediated lung disease. *FASEB J.* 21 1026–1036. 10.1096/fj.06-7301com 17227950

[B48] O’Hara A. M. Shanahan F. (2006). The gut flora as a forgotten organ. *EMBO Rep.* 7 688–693. 10.1038/sj.embor.7400731 16819463 PMC1500832

[B49] Opal S. M. (2007). The host response to endotoxin, antilipopolysaccharide strategies, and the management of severe sepsis. *Int. J. Med. Microbiol.* 297 365–377. 10.1016/j.ijmm.2007.03.006 17452016

[B50] Park K. S. Choi K. H. Kim Y. S. Hong B. S. Kim O. Y. Kim J. H. (2010). Outer membrane vesicles derived from *Escherichia coli* induce systemic inflammatory response syndrome. *PLoS One* 5:e11334. 10.1371/journal.pone.0011334 20596524 PMC2893157

[B51] Park K. S. Lee J. Jang S. C. Kim S. R. Jang M. H. Lötvall J. (2013). Pulmonary inflammation induced by bacteria-free outer membrane vesicles from *Pseudomonas aeruginosa*. *Am. J. Respir. Cell Mol. Biol.* 49 637–645. 10.1165/rcmb.2012-0370OC 23713467

[B52] Peters K. Unger R. E. Brunner J. Kirkpatrick C. J. (2003). Molecular basis of endothelial dysfunction in sepsis. *Cardiovasc. Res.* 60 49–57. 10.1016/S0008-6363(03)00397-3 14522406

[B53] Santos J. C. Dick M. S. Lagrange B. Degrandi D. Pfeffer K. Yamamoto M. (2018). LPS targets host guanylate-binding proteins to the bacterial outer membrane for non-canonical inflammasome activation. *EMBO J.* 37:e98089. 10.15252/embj.201798089 29459437 PMC5852652

[B54] Shimada H. Rajagopalan L. E. (2010). Rho kinase-2 activation in human endothelial cells drives lysophosphatidic acid-mediated expression of cell adhesion molecules via NF-kappaB p65. *J. Biol. Chem.* 285 12536–12542. 10.1074/jbc.M109.099630 20164172 PMC3282996

[B55] Shimazu R. Akashi S. Ogata H. Nagai Y. Fukudome K. Miyake K. (1999). MD-2, a molecule that confers lipopolysaccharide responsiveness on Toll-like receptor 4. *J. Exp. Med.* 189 1777–1782. 10.1084/jem.189.11.1777 10359581 PMC2193086

[B56] Smith W. B. Gamble J. R. Clark-Lewis I. Vadas M. A. (1991). Interleukin-8 induces neutrophil transendothelial migration. *Immunology* 72 65–72. 1997402 PMC1384337

[B57] Somerville J. E. Jr. Cassiano L. Darveau R. P. (1999). *Escherichia coli* msbB gene as a virulence factor and a therapeutic target. *Infect. Immun.* 67 6583–6590. 10569778 10.1128/iai.67.12.6583-6590.1999 PMC97070

[B58] Srisatjaluk R. Doyle R. J. Justus D. E. (1999). Outer membrane vesicles of *Porphyromonas gingivalis* inhibit IFN-gamma-mediated MHC class II expression by human vascular endothelial cells. *Microb. Pathog.* 27 81–91. 10.1006/mpat.1999.0287 10458919

[B59] Untergasser A. Cutcutache I. Koressaar T. Ye J. Faircloth B. C. Remm M. (2012). Primer3–new capabilities and interfaces. *Nucleic Acids Res.* 40:e115. 10.1093/nar/gks596 22730293 PMC3424584

[B60] Wagner J. G. Roth R. A. (2000). Neutrophil migration mechanisms, with an emphasis on the pulmonary vasculature. *Pharmacol. Rev.* 52 349–374. 10977867

[B61] Zhang H. Peterson J. W. Niesel D. W. Klimpel G. R. (1997). Bacterial lipoprotein and lipopolysaccharide act synergistically to induce lethal shock and proinflammatory cytokine production. *J. Immunol.* 159 4868–4878. 9366412

[B62] Zhou H. Andonegui G. Wong C. H. Kubes P. (2009). Role of endothelial TLR4 for neutrophil recruitment into central nervous system microvessels in systemic inflammation. *J. Immunol.* 183 5244–5250. 10.4049/jimmunol.0901309 19786543

