# Supplementary material for: Outer Membrane Vesicles Derived From Escherichia coli Regulate Neutrophil Migration by Induction of Endothelial IL-8
Source: Front Microbiol. 2018 Oct 11;9:2268. doi: 10.3389/fmicb.2018.02268 (PMC6194319; doi:10.3389/fmicb.2018.02268)
Supplement: Supplementary file 1 [file Table_1.DOCX]

Supplementary Material

Outer Membrane Vesicles Derived from *Escherichia coli* Regulate Neutrophil Migration by Induction of Endothelial IL-8

Jaewook Lee*, Yae Jin Yoon, Ji Hyun Kim, Nhung Thi Hong Dinh, Gyeongyun Go, Sookil Tae, Kyong-Su Park, Hyun Taek Park, Changjin Lee, Tae-Young Roh, Dolores Di Vizio, and Yong Song Gho*

*** Correspondence:** Yong Song Gho: ysgho@postech.ac.kr

Jaewook Lee: jaewook8@postech.ac.kr

**Supplementary Figures**

**
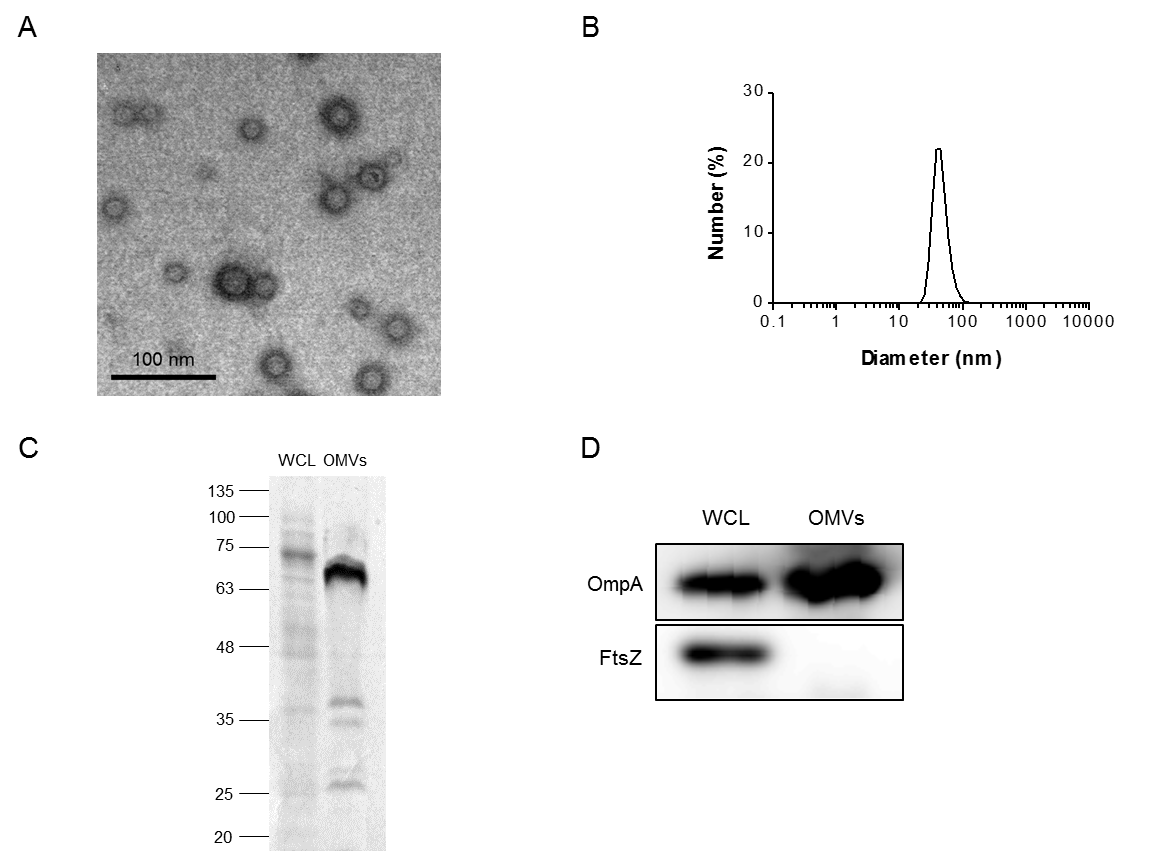
**

**Supplementary Figure 1. Characterization of *E. coli* OMVs.** (A) Transmission electron microscopy indicating the vesicular structures of the purified OMVs. (B) Dynamic light scattering indicating the size distribution of *E. coli* OMVs, ranging 20-100 nm in diameters. (C) Coomassie Brilliant Blue staining of whole cell lysates (WCL) and OMVs, each 5 μg in total protein amounts. Molecular weight standards are indicated on the left (kDa). (D) Western blot directed against OmpA and a cytosolic protein FtsZ. A total of 1 or 5 μg of protein samples from WCL and OMVs were loaded to detect OmpA or FtsZ, respectively.

**
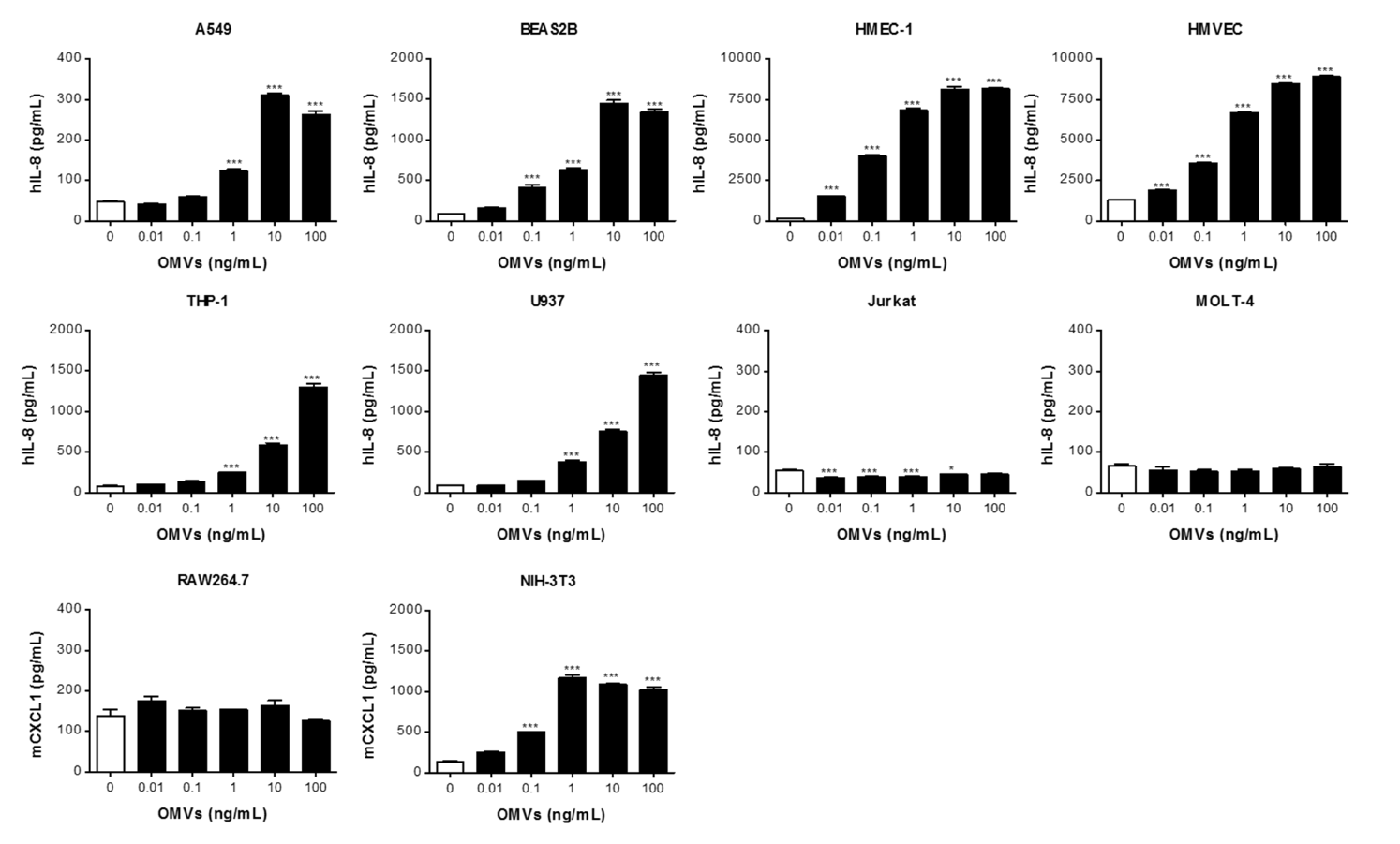
**

**Supplementary Figure 2.** **Induction of IL-8 or CXCL1 from diverse cell lines by *E. coli* OMVs.** *E. coli* OMVs (0, 0.01, 0.1, 1, 10, and 100 ng/mL in total protein concentrations) were treated to diverse cell lines for 12 hours: epithelial cells (A549 and BEAS2B), endothelial cells (HMEC-1 and HMVEC), monocytes (THP-1 and U937), T cells (Jurkat and MOLT-4), macrophages (RAW264.7), and fibroblasts (NIH-3T3). The concentrations of human IL-8 or mouse CXCL1 were measured in the culture supernatants of human (A549, BEAS2B, HMEC-1, HMVEC, THP-1, U937, Jurkat, and MOLT-4) and mouse cells (RAW264.7 and NIH-3T3), respectively, by ELISA (n = 3). Data were represented as mean ± SEM. *, P < 0.05 and ***, P < 0.001, calculated by one-way ANOVA with Bonferroni correction for multiple comparisons.

**
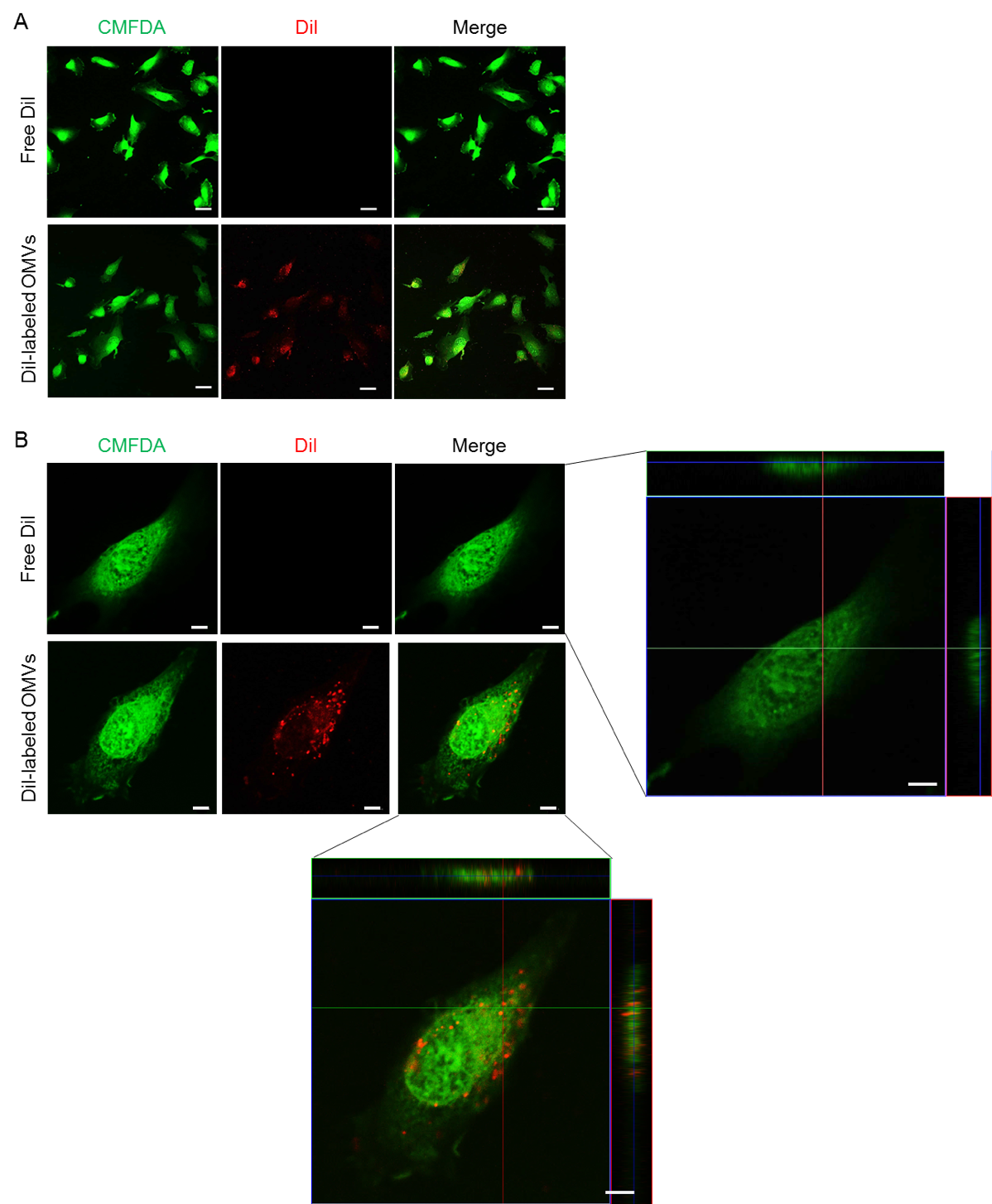
**

**Supplementary Figure 3. Uptake and internalization of *E. coli* OMVs.** Uptake and internalization of free DiI or DiI-labeled *E. coli* OMVs (red fluorescent signal) by HMEC-1 labeled with 5-chloromethylfluorescein (CMFDA, green fluorescent signal) was examined using a confocal microscopy. (A) Low magnificent two-dimensional fluorescence images. Scale bars = 30 μm. (B) High magnificent two-dimensional fluorescence images with representative three-dimensional fluorescence images. Scale bars = 5 μm. Note that free DiI control or DiI-labeled *E. coli* OMVs were prepared by a size-exclusion spun column to remove residual DiI: free DiI control itself does not contain any fluorescent signal.


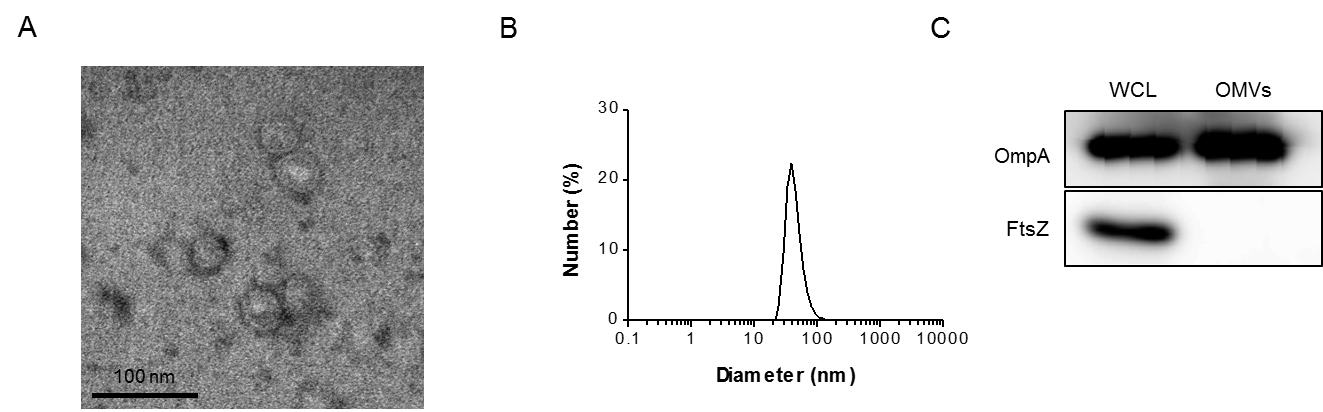


**Supplementary Figure 4. Characterization of *E. coli* OMVs : BDG.** (A) Transmission electron microscopy indicating the vesicular structures of the purified OMVs. (B) Dynamic light scattering indicating the size distribution of *E. coli* OMVs, ranging 20-100 nm in diameters. (C) Western blot directed against OmpA and a cytosolic protein FtsZ. A total of 1 or 5 μg of protein samples from WCL and OMVs were loaded to detect OmpA or FtsZ, respectively

**Supplementary Tables**

**Supplementary Table 1. Primers used for real time RT-PCR.**

| **Gene symbol** | **Forward/reverse** | **Sequences** |
| --- | --- | --- |
| GAPDH | Forward | 5’-CGAGATCCCTCCAAAATCAA-3’ |
|  | Reverse | 5’-TTCACACCCATGACGAACAT-3’ |
| TLR1 | Forward | 5’-GCTGTTCAGCTCTTCTGTTT-3’ |
|  | Reverse | 5’-TGCCTTTGTTATCCTGATTT-3’ |
| TLR2 | Forward | 5’-GGCCAACTGTAATCTGTAGC-3’ |
|  | Reverse | 5’-AGCAGGAAGAAAGAATGACA-3’ |
| TLR3 | Forward | 5’-TTCAGGAACCTGACTGAGTT-3’ |
|  | Reverse | 5’-TGGTATGGGTCTCGTTAATC-3’ |
| TLR4 | Forward | 5’-TCAGAGTGTGTTTGGTTTGA-3’ |
|  | Reverse | 5’-GCACAAATGCACACATCTAC-3’ |
| TLR5 | Forward | 5’-CGTATCAACACCATGTTCTG-3’ |
|  | Reverse | 5’-GAAAATCAATGGAGACTGGA-3’ |
| TLR6 | Forward | 5’-GTTAGCCTGCCAGTTAGAGA-3’ |
|  | Reverse | 5’-GAGCTGAAAAACTGAGCATT-3’ |
| TLR7 | Forward | 5’-GATTGCAAAAATCCAAGAAG-3’ |
|  | Reverse | 5’-GAAATGGGGCATTATAACAA-3’ |
| TLR8 | Forward | 5’-GACACTGCTGCTGAGTCATA-3’ |
|  | Reverse | 5’-GCAGATTGGAACTTAAATCG-3’ |
| TLR9 | Forward | 5’-CTGGACCTGAGTGAGAACTT-3’ |
|  | Reverse | 5’-TTTGGTAATTGAAGGACAGG-3’ |
| CD14 | Forward | 5’-TCTGCAGTAGAGGTGGAGAT-3’ |
|  | Reverse | 5’-CTTGACCGTGTCAGCATAC-3’ |
| MD2 | Forward | 5’-CCAAAGGATTATTGCACATT-3’ |
|  | Reverse | 5’-CTTTGGAAGATTCATGGTGT-3’ |
| NOD1 | Forward | 5’-TCCAAAGCCAAACAGAAACTC-3’  5’-CAGCATCCAGATGAACGTG-3’ |
|  | Reverse |  |
| NOD2 | Forward | 5’-GAAGTACATCCGCACCGAG-3’ |
|  | Reverse | 5’-GACACCATCCATGAGAAGACAG-3’ |
